# NURSES’ PERSPECTIVES ON MEDICATION ADMINISTRATION SAFETY FOR RESIDENTS IN NURSING HOMES: A QUALITATIVE STUDY

**DOI:** 10.1093/geroni/igae098.3872

**Published:** 2024-12-31

**Authors:** Bianca Shieu, Mei-Chen Wang, John Harris, Ya-Wen Lee

**Affiliations:** UT Health San Antonio, San Antonio, Texas, United States; Zoe Liu & Eric Zhou Medical Office PC, New York, New York, United States; Magee-Womens Research Institute, Pittsburgh, Pennsylvania, United States; Changhua Christian Hospital, Changhua, Changhua, Taiwan (Republic of China)

## Abstract

Medication administration (MA) in nursing homes (NH) offers several key benefits to residents. However, the complexity of MA often overwhelms existing systems and individual capacities, leading to dissatisfaction among NH residents and staff. Enhancing safety and efficacy requires a robust, user-centered evaluation of the NH MA system, focusing on safety, efficiency, and staff satisfaction issues and opportunities. This qualitative study examined NH staff nurse perspectives regarding MA safety concerns and potential solutions for NH residents, including those with Alzheimer’s Disease and Related Dementias (ADRD). We used purposeful sampling to recruit 12 nurses employed at two NHs in the northeastern United States to participate in phone interviews. The Systems Engineering Initiative for Patient Safety (SEIPS) constructs informed the question guide, coding, and qualitative theme identification. We audio-recorded and transcribed the interviews verbatim. We organized the themes based on SEIPS components, which include (1) Technology-related concerns (non-user-friendly charting systems). (2) Organization-related concerns (teamwork, communication, and leadership). (3) Person-related concerns (variability in staff training and backgrounds). (4) Task-related concerns (different care approaches for ADRD residents, extreme workload variation throughout the day, task prioritization). (5) Environment-related concerns (staff shortages). Overall, the key themes identified by participants included the significance of user-friendly charting systems, customizing MA to accommodate diverse nursing backgrounds, and appropriate nurse-to-patient staffing ratios.

